# Sea-Sickness

**Published:** 1901-07-13

**Authors:** 


### SEA-SICKNESS.

In the many discussions which have taken place from time to time as to the cause of sea-sickness a strong tendency has manifested itself for the disputants to take one-sided views. Some have maintained that the whole affection was an affair of vision, or of disturbance of equilibrium in the semi-circular canals, or of derangement of the circulation in the brain, while not a few practically-minded people, with their eyes fixed upon the final product as seen in the steward's basin, have held that bile is always at the bottom of the mischief. The truth is that no one of these explanations covers the entire field, while no one of them can be entirely neglected, and that sea-sickness is the summation of the effect of the motion of the ship upon a number of sensitive organs—in fact, upon the whole body. Some of these effects we cannot eliminate, some we can. By lying down we can to a certain extent lessen the effect upon the cerebral circulation, by shutting the eyes we can eliminate the effect on the visual centre, and by carefully swaddling the abdomen in a long and wide flannel bandage, one can to a considerable degree lessen the internal swashing of the intestinal contents. There is another point, however, to be considered, and that is the condition of the abdominal viscera at the time of starting. Dr. James Wortobet[1] tells us that he has travelled more than 100,000 nautical miles, and has usually had under his care several hundred passengers beside the crew. He therefore speaks from experience when he says that although there may be certain cases which are of cerebral origin, such cases are in the minority, and that in the majority of cases the symptoms start from the abdomen. People who are well inured to sea life and are usually quite free from sickness, may still suffer if they go to sea with loaded bowels, and he is quite sure that by the precautions often taken by experienced travellers they do, in fact, protect themselves from sea-sickness which would otherwise occur, such precautions being the taking of a saline purgative the day or so before travelling, adopting the recumbent posture, and avoiding oleaginous smells and the company of those who are sea-sick. He strongly advises those who suffer principally from gastric phenomena to provide themselves with a good flannel bandage, twelve feet long and six inches broad, and wind it round their trunk over the whole width of the abdominal region. This will afford great comfort by preventing the contents of the abdomen viscera from undue movements. He also says that for severe retching and persistent sickness nothing is so trustworthy as a hypodermic injection of morphine.

[1] Brit. Med. Jour., June 29.

